# Quantitative PCR for detection of the OT-1 transgene

**DOI:** 10.1186/1471-2172-6-20

**Published:** 2005-08-24

**Authors:** Kate O Wright, Debbie A Murray, Nicholas I Crispe, Robert H Pierce

**Affiliations:** 1 Department of Pathology, University of Rochester Medical Center, Rochester, NY 14642, USA; 2 David H. Smith Center for Vaccine Biology and Immunology, Aab Institute of Biomedical Sciences, University of Rochester, Rochester, NY 14642, USA

## Abstract

**Background:**

Transgenic TCR mice are often used experimentally as a source of T cells of a defined specificity. One of the most widely used transgenic TCR models is the OT-1 transgenic mouse in which the CD8+ T cells express a TCR specific for the SIINFEKL peptide of ovalbumin presented on k^b^. Although OT-1 CD8+ can be used in a variety of different experimental settings, we principally employ adoptive transfer and peptide-driven expansion of OT-1 cells in order to explore the distribution and fate of these antigen-specific OT-1 T cells. We set out to develop a quantitative PCR assay for OT-1 cells in order to assess the distribution of OT-1 CD8+ T cells in tissues that are either intrinsically difficult to dissociate for flow cytometric analysis or rendered incompatible with flow cytometric analysis through freezing or fixation.

**Results:**

We show excellent correlation between flow cytometric assessment of OT-1 cells and OT-1 signal by qPCR assays in cell dilutions as well as in *in vivo *adoptive transfer experiments. We also demonstrate that qPCR can be performed from archival formalin-fixed paraffin-embedded tissue sections. In addition, the non-quantitative PCR using the OT-1-specific primers without the real-time probe is a valuable tool for OT-1 genotyping, obviating the need for peripheral blood collection and subsequent flow cytometric analysis.

**Conclusion:**

An OT-1 specific qPCR assay has been developed to quantify adoptively transferred OT-1 cells. OT-1 qPCR to determine cell signal is a valuable adjunct to the standard flow cytometric analysis of OT-1 cell number, particularly in experimental settings where tissue disaggregation is not desirable or in tissues which are not readily disassociated

## Background

Adoptive transfer of CD8+ T cells from MHC Class I-restricted OVA specific T cell receptor (TCR) transgenic mice (OT-1) into host C57BL/6 mice allows for the activated CD8+ T cell population to be tracked and quantified after stimulation with the OVA peptide SIINFEKL [1] using standard flow cytometric analysis. Using this methodology, it has been demonstrated that activated CD8+ T cells undergo migration to the liver where they are trapped and undergo apoptosis [2]. While flow cytometry is sensitive and specific, it is best suited to tissues from which single cell suspensions can be obtained easily, such as the spleen and peripheral lymph nodes. Although flow cytometry can be performed on lymphocytes isolated from other tissues such as liver, muscle and intestine, it is technically more challenging and questions may be raised regarding whether the cell sample is representative of the in vivo state. Reports by Lang (1997), Lim (2002), and Gallard (2002), [3-5] also emphasize these points and describe PCR based approaches for tracking specific T-cell clonotypes. Additionally, sample preparation for histologic analysis and nucleic acid or protein-based assessment is incompatible with tissue dis-aggregation methods for flow cytometry. In order to augment standard flow cytometric analysis in these more challenging experimental settings, a real-time quantitative PCR assay was developed to quantify and track activated OT-1 cells with a high degree of sensitivity.

Because the OT-1 mouse was made by micronuclear injection of genomic DNA, which did not contain unique transgene non-coding sequence, we based a quantitative PCR strategy on the DNA sequence encoding the rearranged VDJ sequences corresponding to the CDR3 region of the TCR Beta chain. As genomic DNA is the starting point for qPCR, any tissue harvested from a host mouse can be assayed for the OT-1 transgene. Additionally, nucleic acid isolated from paraffin-embedded material is also a suitable template for real-time quantitative PCR, making this technique useful for analysis of archival paraffin-embedded material.

The utility and sensitivity of a qPCR assay designed to detect the OT-1 transgene was compared to the standard method of flow cytometry to track and quantify activated OT-1 cells in both in vitro dilution and in vivo adoptive transfer experiments.

## Results

### OT-1 Genotyping PCR is a specific and reliable method to determine positive OT-1 mice from wild-type littermates

**Figure 1:**
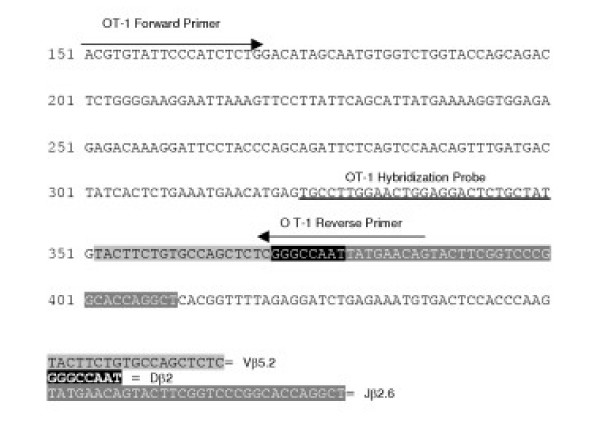
**Schematic Representation of Primers and Probes. **Schematic representation of primers and hybridization probe location within the VDJ region of the OT-1 T Cell Receptor Beta chain.

**Figure 2:**
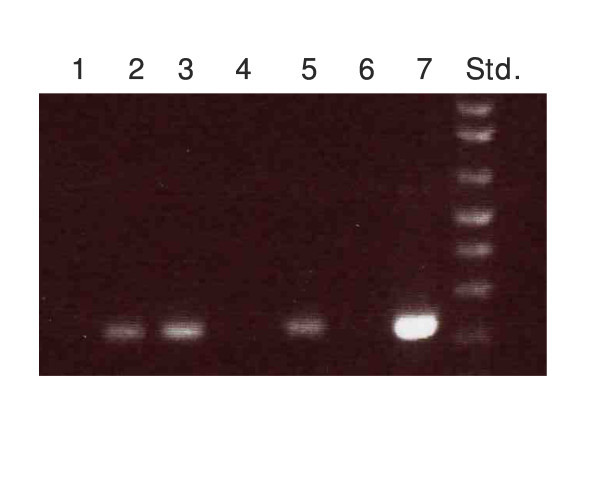
**OT-1 Genotyping Results. **OT-1 genotyping results demonstrate the 238 bp OT-1 fragment was amplified from OT-1 positive transgenic mice (lanes 2, 3, and 5) but not from negative littermates (lanes 1, 4, and 6). Plasmid pcRTOPO2.1 containing the cloned OT-1 product was used as a positive control (lane 7).

A PCR strategy was developed to identify OT-1 positive transgenic mice, which express a T cell receptor (TCR) specific for the OVA peptide SIINFEKL, from wild-type littermates. Primers were designed in the VDJ region of the OT-1 TCRβ chain (Figure 1) to yield a 238 bp product. Figure 2 demonstrates that the OT-1 fragment is amplified from OT-1 positive transgenic mice (lanes 2, 3, and 5), but not from wild-type littermates (lanes 1,4, and 6). Flow cytometric phenotyping of PCR positive OT-1 mice confirmed the specificity of the reaction (data not shown).

**Figure 3:**
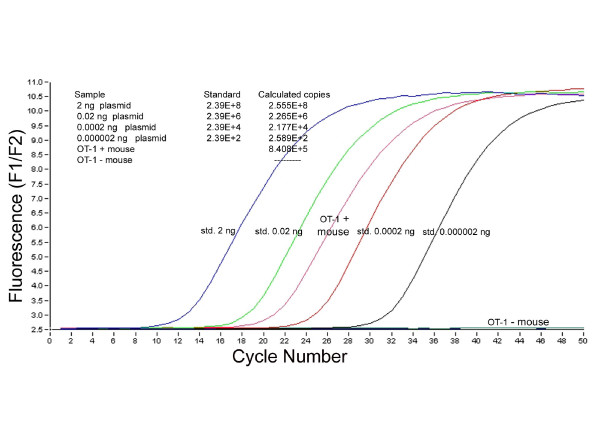
**Quantitative Real-time PCR Data**. Quantitative real-time PCR data for the OT-1 transgene using pCR TOPO2.-OT-1 plasmid standards and genomic DNA from OT-1 positive and OT-1 negative mice. 8.408 *10^5 ^copies of the OT-1 transgene were detected in 300 ng of genomic DNA from the OT-1 positive mouse while no signal was generated from the OT-1 negative mouse.

A quantitative real-time PCR assay was developed using a 27 base pair fluorophore-coupled oligonucleotide probe, with sequence-dependent hybridization within the 238 base pair amplified VDJ region of the OT-1 TCR. This assay was successful at determining OT-1 transgenic mice from negative littermates. Figure 3 shows data from a real-time PCR experiment using the pCRTOPO2.1-OT1 plasmid at various concentrations and genomic DNA isolated from positive and negative OT-1 transgenic mice. Based on the plasmid standard curve, 8.408E+5 copies of the OT-1 transgene were present in 300 ng of genomic DNA from an OT-1 positive mouse. There was no signal amplification when genomic DNA from the OT-1 negative mouse was used as template, or in the no template control. To normalize all qPCR results for the OT-1 transgene, a fluorophore-coupled oligonucleotide probe and primer set for murine β-actin was also designed and used for each sample.

To improve upon the sensitivity of the quantitative PCR assay sequence-independent detection with SYBR Green I was also performed. Double-stranded DNA product was quantified by monitoring fluorescence of the DNA-binding SYBR Green I dye. Plasmid pCRTOPO2.1 containing the OT-1 fragment was used as template in a standard curve analysis at concentrations of 2 ng to 2 E-8 ng, correlating to 4.38E+8 to 4.38E+0 copies of transgene. This real-time PCR strategy was successful at detection down to 4.38 copies of the transgene and did not amplify signal in the absence of DNA template. Melting curve analysis also showed a single PCR product was generated. In subsequent experiments using genomic DNA isolated from animal tissue, however, high background signals and multiple fluorescent peaks were generated, indicating a quantitative PCR assay based on SYBR Green would not be sufficient for future experiments.

### Sensitivity and specificity of real-time quantitative PCR on diluted OT-1 genomic DNA

**Figure 4:**
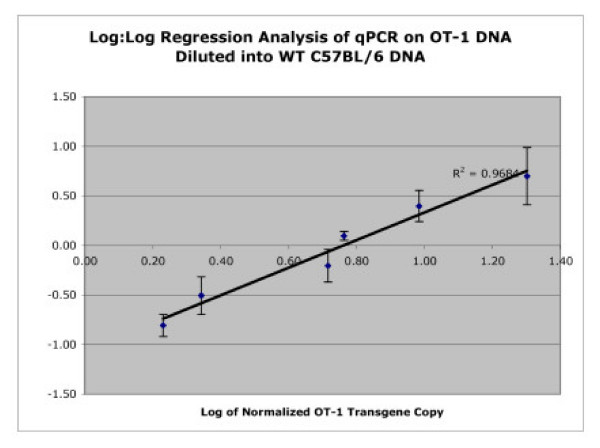
**Regression Analysis of OT-1 Copy Number. **Regression analysis of the logarithmic values for normalized OT-1 copy number (defined as the number of OT-1 copies/10,000 β-actin copies) by real-time qPCR (done in triplicate) plotted against the logarithmic values of the actual DNA dilution values. The R^2 ^value was calculated to be 0.9684.

The linear range of sensitivity of the qPCR assay was determined by diluting OT-1 DNA (isolated from the liver of an OT-1 transgenic mouse) with genomic DNA isolated from a non-transgenic C57BL/6 normal mouse. Figure 4 shows a regression analysis of the logarithmic values for normalized OT-1 copy number (defined as the number of OT-1 copies/10,000 β-actin copies) by real-time qPCR (done in triplicate) plotted against the logarithmic values of the actual DNA dilution values. The calculated R^2 ^value for the regression analysis was 0.9684. The real-time assay was used to quantify a range of 5% to 0.1560% of OT-1 DNA diluted into background C57BL/6 DNA. The real-time qPCR could also distinguish 0.3125% OT-1 DNA from 0.1560% OT-1 DNA, demonstrating that the sensitivity and specificity of the assay would be sufficient for future applications.

### Quantitative real-time PCR for detection of OT-1 CD8+T cells diluted into normal C57Bl/6 splenocytes

To test the sensitivity of the real-time assay, OT-1 CD8 +T cells were purified from an OT-1 transgenic mouse. OT-1 cells were diluted into splenocytes isolated from normal C57BL/6 mice so that the dilutions were comprised of 100%, 4%, 1,3%, 0.433%, 0.144%. 0.048%, and 0.016% OT-1 cells in a background of wild-type C57BL/6 splenocytes. Previous to the dilutions, flow cytometric analysis was performed to determine cell purity and yield of both cell suspensions, which were both resuspended at 8 million cells/mL. 200 μL was taken for flow cytometric analysis and genomic DNA was isolated from the remaining 3.2 million cells. 300 ng of genomic DNA from each dilution (done in triplicate) was used as template for quantitative PCR to determine OT-1 and β-actin copy number.

**Figure 5:**
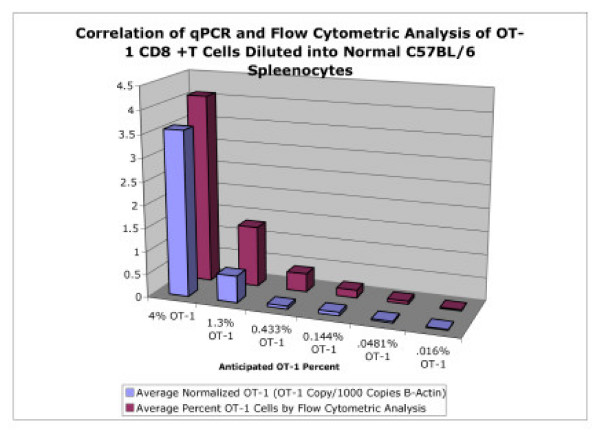
**Correlation of qPCR and Flow Cytometry**. Correlation of qPCR and flow cytometric analysis of for detection of OT-1 cells after dilution into wild-type C57BL/6 spleenocytes. The cell dilutions were calculated to represent 4%, 1,3%, 0.433%, 0.144%. 0.048%, and 0.016% total OT-1 CD8+T cells.

**Figure 6:**
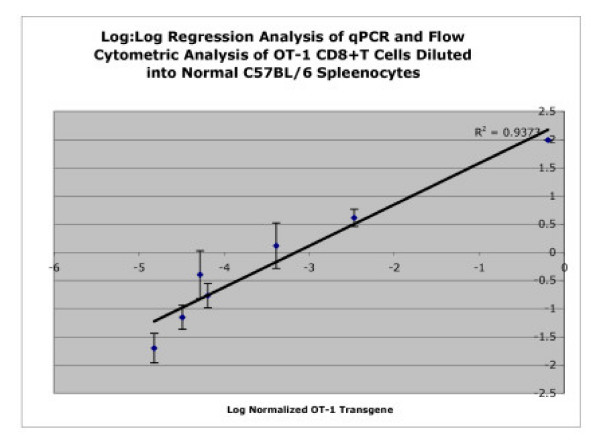
**Regression Analysis of qPCR and Flow Cytometry. **Regression analysis for the logarithmic values of normalized OT-1 transgene copy (defined as the number of OT-1 copies/10,000 β-actin copies) and actual percentage of OT-1 cells by flow cytometry. OT-1 dilutions of 100%, 4%, 1,3%, 0.433%, 0.144%. 0.048%, and 0.016% were used for the analysis and the R^2 ^value was calculated to be 0.9458.

Figure 5 demonstrates the correlation between the results obtained from qPCR for the average normalized OT-1 transgene copy number and the average actual percentages of OT-1 cells determined by flow cytometry. To further analyze the data and to separate the cluster of data points, regression analysis for the logarithmic values of normalized OT-1 transgene copy (defined as the number of OT-1 copies/10,000 β-actin copies) and actual percentage of OT-1 cells by flow cytometry was done. The OT-1 dilutions of 100%, 4%, 1,3%, 0.433%, 0.144%. 0.048%, and 0.016% were used in the analysis. Figure 6 demonstrates the regression analysis with a calculated R^2^value of 0.9458 and also shows that linearity of the assay was maintained and the lowest dilution values of 0.048% versus 0.016% could be resolved. Again, the sensitivity and specificity of the real-time assay are evidence for the utility of qPCR for future applications.

### Accumulation of activated OT-1 T-cells after adoptive transfer by real-time quantitative PCR

Accumulation of activated OT-1 CD8+ T cells in the liver after adoptive transfer and in vivo activation and expansion has been well documented [6-8]. The purpose of these experiments was to determine whether a quantitative PCR assay would be sensitive enough to also monitor and quantify the migration of T-cells to the liver and other organs after an adoptive transfer experiment compared to results obtained using standard flow cytometric analysis.

**Figure 7:**
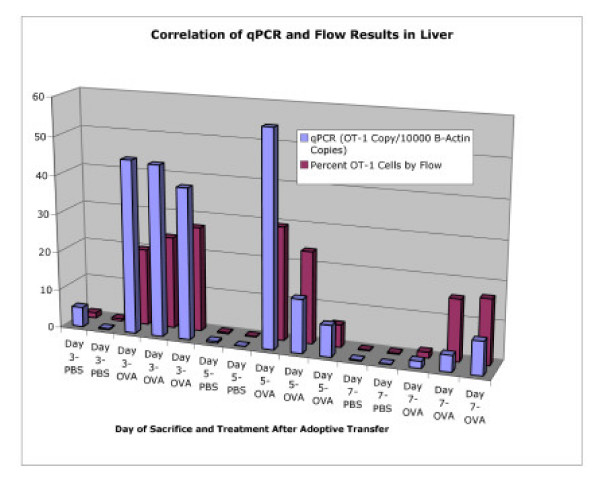
**Correlation of qPCR and Flow Cytometry After Adoptive Transfer. **Correlation between qPCR and flow cytometry for detection and quantification of OT-1 present in the livers of host animals after adoptive transfer. Mice were sacrificed on days 3, 5, or 7 after the first i.p. injection of stimulating OVA peptide, or PBS control.

Figure 7 demonstrates the correlation between flow cytometric analysis and qPCR to quantify OT-1 cells in the liver of animals given i.p. injections of PBS or OVA stimulating peptide. Livers were harvested on days 3, 5, and 7 after the first i.p. injection of peptide. One lobe was reserved for DNA isolation and qPCR, the remainder of the tissue was prepared for flow cytometric analysis. The percent of OT-1 cells by flow cytometric analysis were compared to the OT-1 gene levels (shown as the number of OT-1 gene copies per 1*10^5 ^copies of β-actin). Although there is variation in the absolute numbers, both methods demonstrate an expansive OT-1 CD8+ T-cell population in the liver of OVA treated animals, which diminishes by the day 7 timepoint.

**Figure 8:**
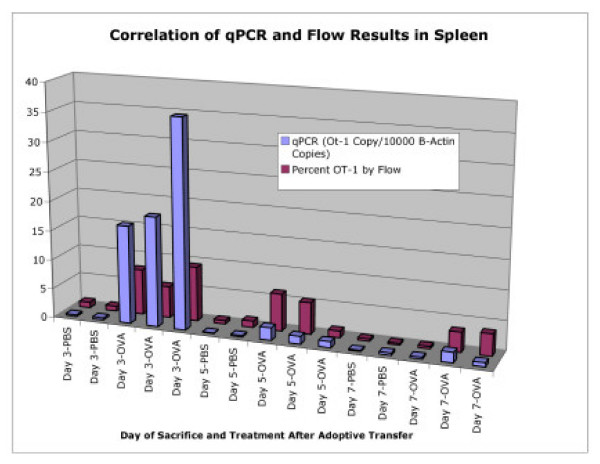
**Correlation of qPCR and Flow Cytometry After Adoptive Transfer. **Correlation between qPCR and flow cytometry for detection and quantification of OT-1 present in the spleens of host animals after adoptive transfer. Mice were sacrificed on days 3, 5, or 7 after the first i.p. injection of stimulating OVA peptide, or PBS control.

Spleens from the same animals, sacrificed on days 3, 5, and 7 after first i.p. injection with peptide, were analyzed in the same manner (Figure 8). Roughly half of the spleen was prepared for flow cytometric analysis and the remaining half was used for DNA isolation and qPCR. The percent of OT-1 cells by FACS analysis in the spleen samples were compared to the normalized OT-1 gene levels. Again, while there was variation in the absolute numbers, both methods show an expanded OT-1 CD8+ T-cell population in the spleens of OVA treated animals, which is most prominent on the day 3 timepoint, and had similar trends. When the normalized copies of the OT-1 transgene for all liver and spleen samples from each timepoint were plotted against the percent of OT-1 cells by flow cytometric analysis, the correlation coefficient was calculated at R^2 ^= 0.7027.

The results from these experiments demonstrate the utility of a quantitative PCR assay on a tissue that is amenable (spleen) and one that is not so amenable (liver) to flow cytometry. While the trends of OT-1 cell accumulation in the various organs were very similar there were definite differences in the resulting numbers. Part of the discrepancy may be due to sampling artifact: Although the same tissue specimens were used for flow cytometry and qPCR, different sections of the tissue were used in each assay. A small portion of tissue (approximately 75 mg) was needed for DNA isolation, while one half to three-quarters of the tissue was used to make cell suspensions for flow cytometry, implying that variation inherent to each individual organ was present and was a possible contributor to the observed discrepancies. Also pertinent to this argument is to point out that flow cytometric analysis in the liver involves sampling the leukocyte fraction after a mononuclear cell isolation, where DNA isolation for qPCR analysis from whole liver includes the large parenchymal population. Quantitative PCR and flow cytometry also have different denominators to consider. Flow cytometry determines the percent of OT-1 cells out of the selected CD8+ CD45.1+ positive population in the leukocyte gate while qPCR determines the number of OT-1 copies in all of the sampled DNA.

**Figure 9:**
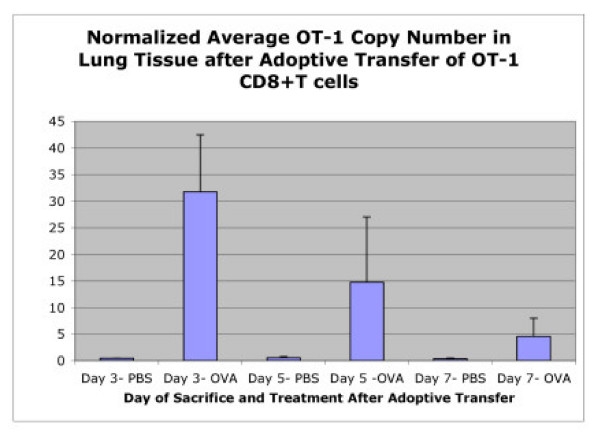
**Detection of OT-1 Cells in Lungs After Adoptive Transfer. **Quantitative PCR for OT-1 transgene demonstrates migration of activated OT-1 cells into the lungs of host animals after adoptive transfer. The normalilzed OT-1 transgene copy numbers were averaged for each treatment (PBS or stimulating OVA peptide) on mice sacrificed on days 3, 5, or 7 after the first i.p. injection was administered.

To further test the utility of this assay, the lungs of each animal were also flash frozen and stored for later DNA isolation. Due to the difficulty in obtaining adequate materiel for single cell suspensions, flow cytometric analysis was not performed from lung tissue. Since qPCR requires isolated nucleic acid material and not single cell suspensions, quantitative OT-1 transgene numbers could be easily obtained from lung samples. After normalization to the β-actin control, results from quantitative PCR show a marked increase in OT-1 transgene copy number in the OVA treated animals compared to PBS controls (Figure 9). These results further demonstrate the utility of this assay for tissue samples that are difficult to disassociate for flow cytometry and also that a migratory OT-1 CD8+ T cell population is present in lung tissue of animals after adoptive transfer experiments.

### Quantitative PCR analysis of OT-1 transgene on archival material

**Figure 10:**
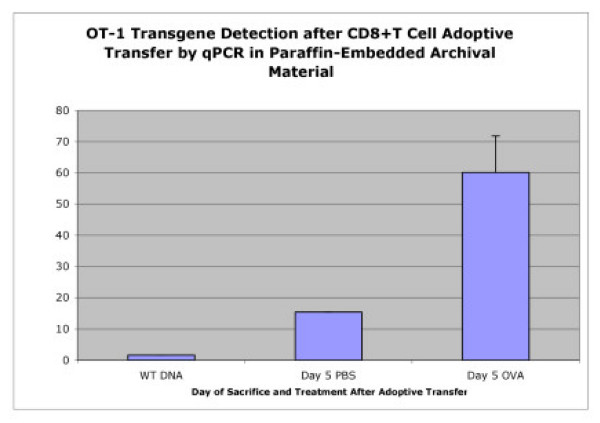
**Detection of OT-1 Cells from Archival Material. **Detection of OT-1 cells from paraffin-embedded archived material is possible using the OT-1 qPCR assay. Paraffin embedded liver sections (5 uM) of C57BL/6 mice used as hosts in OT-1 CD8+ T cell adoptive transfer experiments were used to generate template DNA. The animals were sacrificed on day five after the first i.p injection of the stimulating OVA peptide or PBS control.

Paraffin embedded slides (5 uM) were made from liver sections of C57BL/6 mice used as hosts in OT-1 CD8+ T cell adoptive transfer experiments. The animals were sacrificed on day five after the first i.p injection of the stimulating OVA peptide or PBS control. Histological examination of the slides confirmed the presence of foci (trapped and apoptotic T cells) in the livers of animals given OVA peptide while livers of mice given PBS injections did not have significant foci formation. DNA isolated from the paraffin-embedded slides was used in real-time qPCR for detection of the OT-1 transgene. Although the paraffin slides had been stored at room temperature for approximately 18 months, genomic DNA was readily isolated from the samples. Figure 10 demonstrates average levels of the OT-1 transgene in animals that had received the OVA stimulating peptide were nearly five-fold higher than in PBS control animals, confirming the utility of the qPCR assay on archived experiments.

## Conclusion

The aim of this work was to develop a qPCR method for the tracking and quantification of activated OT-1 CD8+ T cells as an alternative to flow cytometric analysis. While sensitivity and specificity of flow cytometry is sufficient for most applications, it is limited to tissue in which single cell suspensions can be obtained without a high degree of difficulty, such as the spleen and peripheral lymph nodes. Flow cytometry can also be performed on liver tissue, with more difficulty, by using a very time consuming protocol. The purification of mononuclear cells for flow cytometric analysis from brain or muscle tissue is also quite difficult and could limit the value of the transgenic model of T cell activation and clearance in an experimental setting where a complete tissue survey was required. Our real-time quantitative PCR assay is useful for detection of the OT-1 transgene using both *in vitro *and *in vivo *experiments, particularly where flow cytometry is difficult. On a more practical level, it should be noted that quantitative PCR assays are relatively easy to use and can be performed in a variety of settings. Also, samples from ongoing experiments with multiple time points can be harvested and stored until all specimens are obtained and can be prepared all at the same time.

We have validated the quantitative real-time PCR for the OT-1 transgene against known numbers of OT-1 cells in dilution experiments in vitro and correlated the PCR against flow cytometric assessment. In addition, we have validated the OT-1 qPCR against flow cytometric analysis of tissue samples. In all cases, we have observed excellent correlation. Therefore, we are confident that this quantitative PCR based approach to detect OT-1 cells will be a very useful tool in the detection and quantification of OT-1 cells in a variety of experimental models.

## Methods

### Animals

C57B/6J and B6.SJL-*Ptprca Pep3b*/BoyJ mice were obtained from the Jackson Laboratory (Bar Harbor, ME). OT-1 mice express a transgenic TCR that recognizes the 8-mer SIINFEKL peptide derived from residues 257–264 of ovalbumin. Transgenic animals were identified by staining PBL with antibodies against CD8 and the chains of the transgenic TCR, Vα 2 and Vβ 5. A colony of OT-1 mice were maintained on the B6.SJL (CD45.1 expressing) background. All animals were housed in a specific pathogen-free environment in accordance with institutional guidelines for animal care.

### Adoptive transfer and *in vivo *activation

Donor CD8 + OT-1 T cells from mouse spleen and peripheral lymph nodes were purified through depletion of B cells, dendritic cells, NK cells and CD4 +T cells using primary antibodies (clone 212.Al specific for MHC class II molecules, clone 2.4.G2 specific for FcRs, clone GK1.5 specific for CD4 and clone HB.191 specific for NK1.1), followed by magnetic beads (Biomag goat anti-mouse IgM, goat anti-mouse IgG and goat anti-rat IgG, Qiagen). Purity was calculated to be greater than 90%. A suspension of 5 × 10^6 ^OT-1 cells was injected IV into each recipient host mouse. OT-1 T cells were activated after adoptive transfer by daily i.p. injections of 25 nM SIINFEKL peptide (New England Peptide Inc., Fitchburg, MA) in PBS for 3 days starting 24 h after OT-1 transfer as previously described by Mehal, et al [9]. Control mice received an equivalent volume of PBS.

### Mononuclear cell isolation

Intrahepatic lymphocytes were isolated using a standard method (Huang, 1994). In brief, the liver was perfused through the portal vein with 5 ml ice-cold PBS, homogenized by forcing through a metal strainer, then digested with 10 ml of a digestion buffer (RPMI containing 0.02% (w/v) collagenase IV and 0.002% (w/v) DNase I (Sigma, St. Louis, MO)) at 37°C with shaking. The digested liver was centrifuged twice at 10 × g for 4 min at 4°C to remove hepatocytes. The non-parenchymal cells were resuspended to a final volume of 1.4 ml in RPMI before mixing with 2.6 ml of 40% metrizamide (ICN Biomedicals Inc. Aurora, OH) resulting in a final metrizamide concentration of 26%. The cell suspension/metrizamide mixture was then overlayed with 2 ml of RPMI and centrifuged at 1500 × g for 30 min at 4°C. The cells at the interface were collected, washed in HBSS + 5% FBS and counted. Spleens and lymph node cell suspensions were washed at 120 × g for 10 min at 4°C, filtered through 100 μm strainers to remove debris and counted.

### Flow cytometric analysis

Cells were surface stained with Vα2-FITC, Vβ5-PE, CD45.2-FITC, CD45.1-PE, CD8-PerCP (BD Pharmingen) and Vα2-APC (Caltag, Burlingame, CA) for 30 min at 4°C, washed in PBS and fixed in a paraformaldehyde-based fixative. Cells were analyzed using a BD FACSCalibur. Data were analyzed using Cell Quest software (BD Biosciences). OT-1 T cells were identified using light scatter gates characteristic of lymphocytes, as well as positive staining for CD45.1 and CD8.

### DNA isolation from mouse tissue

Tissue sections from donor mice were immediately flash frozen in liquid nitrogen after removal from animal and stored at -85 degrees C until ready for processing. The DNeasy kit (Qiagen) was used to isolated genomic DNA from approximately 25 to 75 mg of flash frozen mouse tissue. Alternatively, to make purified genomic DNA from OT-1 and wild-type C57B6 mice for DNA dilution experiments, whole livers were removed from animals and immediately flash frozen in liquid nitrogen. Tissues were homogenized and digested overnight, at 55 degrees C, in digestion buffer (100 mM NaCl, 10 mM Tric-Cl, pH 8.0, 25 mM EDTA, pH 8.0, 0.5% SDS, and 0.1 mg/mL Proteinase K). Genomic DNA was isolated by phenol:chloroform extraction followed by ethanol precipitation.

### Cell dilutions

CD8 +T cells were isolated from the spleens of two MHC class I-restricted OVA-specific TCR transgenic mouse (OT-1), as described. Monocytes were isolated from the spleens of 5 wild-type C57B6 mice. FACS analysis was performed to determine cell purity and yield, and appropriate cell dilutions were made. 200 uL was taken for further FACS analysis and genomic DNA was isolated from the remaining 3.2 million cells (Qiagen DNeasy kit). 300 ng of genomic DNA from each dilution was used as template for quantitative PCR to determine OT-1 and β-actin copy number.

### Hybridization probe based quantitative PCR

The LightCycler PCR and detection system (Roche Diagnostics) was used for amplification and quantification of the OT-1 transgene from each tissue sample. The LightCycler FastStart DNA Master Hybridization Probes kit was used as described by the manufacturer. Each PCR mixture contained 250 ng of genomic DNA template, 1X LightCycler FastStart reaction buffer, 5.0 mM magnesium chloride, 0.5 uM sense primer (ACGTGTATTCCCATCTCTGG), 0.5 uM antisense primer (CTGTTCATAATTGGCCCGA) and 0.3 uM OT-1 Hybridization probe (5' FAM/TGCCTTGGAACTGGAGGACTCTGCTAT 3'/TAMRA) to amplify 238 bp fragment of the OT-1 transgene. The OT-1 transgene fragment corresponds with nucleotides 150 to 388 of the transgenic T cell receptor. The PCR was performed in glass capillaries with 50 cycles of denaturation (5 s at 95°C) and annealing and extension (75 s at 61°C) after an initial 95°C melt for 10 minutes. Standard curve analysis was performed with each LightCycler run using the OT-1 transgene product cloned into pcCRTOPO2.1 (Invitrogen) for plasmid template. Each DNA sample was also analyzed for β-actin DNA following the same protocol using sense and antisense β-actin primers (agccatgtacgtagccatcc and ttcaccaccacagctgagag), respectively, and a β-actin hybridization probe (5' FAM/ACCTGACGGACTACCTCATGAAGATCC 3'/TAMRA). These primers correspond with mouse genomic DNA on chromosome 5 nucleotide positions 14162633 to 141626562 [10]. Standard curve analysis was performed with each LightCycler run using the mouse β-actin DNA product cloned into pCRBLUNT (Invitrogen) for plasmid template.

### SYBR Green quantitative PCR

To determine sensitivity of a real time PCR assay for OT-1 detection, the LightCycler PCR and detection system (Roche Diagnostics) was used for amplification and quantification of OT-1 DNA. The LightCycler FastStart DNA Master SYBR Green kit was used as described by the manufacturer. Each PCR mixture contained 1X LightCycler reaction buffer, 5 mM magnesium chloride, and 0.5 uM sense (ACGTGTATTCCCATCTCTGG) and antisense (CTGTTCATAATTGGCCCGA) primers to amplify 238 bp fragment of OT-1 transgene. PCR was performed in glass capillaries with 55 cycles of denaturation (2 s at 95°C), annealing (2 s at 61°C), and chain extension (8 s at 72°C). Melting curve analysis from 65°C to 95°C, followed by cooling to 40°C, was also performed. Double-stranded DNA product was quantified by monitoring fluorescence of the DNA-binding SYBR Green I dye. Plasmid pCRTOPO2.1 containing the OT-1 fragment was used as template in standard curve analysis at concentrations of 2 ng to 28*10 ^-8 ^n, correlating to 4.38*10^8 ^to 4.38*10^0 ^copies of transgene.

### DNA isolation from paraffin-embedded slides

C57BL/6 host mice were used in adoptive transfer experiments of OT-1 CD8+T cells, as described. Five days after the first i.p. injection with OVA peptide, animals were sacrificed and livers were fixed in paraffin. Five uM thick slides were cut from experimental livers and also from livers of normal C57BL/6 mice. Paraffin-embedded material was scraped from slides and DNA was isolated following the protocol outlined in the Qiagen QIAmp kit (Invitrogen).

## Authors' contributions

KOW designed and carried out the genotyping and quantitative PCR assays, participated in final analysis and drafted the manuscript. DAM performed the adoptive transfer experiments and flow cytometric analysis. INC participated in design of the study and final data interpretation. RHP participated in design of the study, statistical analysis, and final data interpretation. All of the authors read and approved the final manuscript.
